# SARS Outbreak, Taiwan, 2003

**DOI:** 10.3201/eid1002.030515

**Published:** 2004-02

**Authors:** Ying-Hen Hsieh, Cathy W.S. Chen, Sze-Bi Hsu

**Affiliations:** * National Chung Hsing University, Taichung, Taiwan; † Feng Chia University, Taichung, Taiwan; ‡ National Tsing Hua University, Hsinchu, Taiwan

**Keywords:** severe acute respiratory syndrome, SARS-CoV, infectious diseases, epidemiology, Taiwan, nosocomial infections, quarantine and isolation, prevention and control, theoretical model, effective reproductive number

## Abstract

We studied the severe acute respiratory syndrome (SARS) outbreak in Taiwan, using the daily case-reporting data from May 5 to June 4 to learn how it had spread so rapidly. Our results indicate that most SARS-infected persons had symptoms and were admitted before their infections were reclassified as probable cases. This finding could indicate efficient admission, slow reclassification process, or both. The high percentage of nosocomial infections in Taiwan suggests that infection from hospitalized patients with suspected, but not yet classified, cases is a major factor in the spread of disease. Delays in reclassification also contributed to the problem. Because accurate diagnostic testing for SARS is currently lacking, intervention measures aimed at more efficient diagnosis, isolation of suspected SARS patients, and reclassification procedures could greatly reduce the number of infections in future outbreaks.

On April 22, 2003, the World Health Organization (WHO) reported 3,947 probable severe acute respiratory syndrome (SARS) cases with 229 deaths worldwide (*1*); China, Hong Kong, Singapore, Vietnam, and Toronto, Canada, had the most cases. At that time, Taiwan had 29 probable cases and no deaths. Seventy-eight percent of its cases were imported, and the growth seemed to be exponential but at a comparatively slow rate (*2*), typical of a minor outbreak. A new cluster of seven infections in Hoping Hospital in Taipei was reported on that day (*3*), however, starting a chain of local transmissions that cumulated in 116 probable cases and 10 deaths in a fortnight. In the days that followed, the numbers grew to 264 cases and 34 deaths by mid-May, and 680 cases and 81 deaths by June 1—more than a sixfold increase in <l month.

Many questions arose as to how SARS was able to spread so rapidly in Taiwan, a full 2 months after the global alert posted by WHO and >1 month after its passage through Hong Kong, Singapore, and other neighboring countries (*4*). Inexperience at containing outbreaks and the lack of expert assistance from WHO, at the least at the beginning (*5*), certainly contributed to the problem. So did inadequacies in the health infrastructure, hospital mismanagement, and simple human carelessness. Hsieh and Chen (*2*) observed that the cumulative number of probable cases exhibited seemingly random variations in the period after April 22, a feature that cannot be captured by simple curve-fitting techniques. We studied the waves of infections that occurred in most of May by using a mathematical model tailor-made to the specifics of the SARS outbreak in Taiwan but simple enough to allow researchers to draw inferences.

Riley et al. (*6*) and Lipsitch et al. (*7*) used dynamic models to model the respective transmission dynamics of SARS in Hong Kong and Singapore. The models were complex and general dynamic models, and they allowed researchers to calculate numerous epidemiologically important parameters and assess the potential danger of the epidemic. Many questions remain, however, such as the effect of data quality on results and the role of heterogeneity in disease transmission (*8*). We aimed to circumvent problems in answering these questions with a simple mathematical model useful to our understanding of the outbreak.

## Methods

We proposed a dynamic model to reflect the actual sequence of events for a reported case-patient in Taiwan, from onset to admission at a hospital as a suspected case-patient to either reclassification as a probable case-patient or removal from the suspected SARS category, and finally reclassification from probable case to discharged case or fatality. Our goal was to evaluate the dynamics at work that resulted in rapid epidemic growth during the period observed. We chose to use a discrete difference equation model because the data used are the discrete daily numbers of reported suspected cases, probable cases, and accumulated deaths posted on the Taiwan Center for Disease Control Web site (*9*).

**Figure 1:**
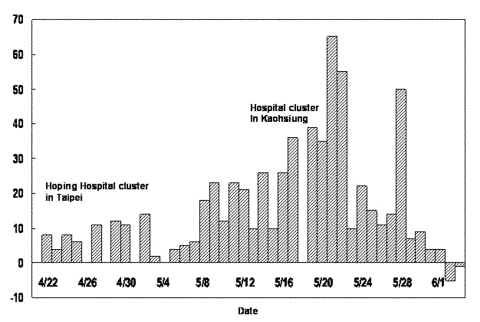
The number of new probable cases in Taiwan by reporting date, May 5–June 4, 2003.

Starting from the Hoping Hospital cluster in Taipei on April 22, the large numbers of cases reported daily (Figure 1) alerted all residents in Taiwan to the danger of SARS, at times to near-panic state. Amid the heightened tension, the health authority tried to enforce stringent measures to contain the outbreak. One measure was reporting, admitting, and hospitalizing all persons suspected of having SARS. Another was the house quarantine of tens of thousands of persons, mainly those with contacts to the suspected case-patients and to arrivals from affected areas abroad. The quarantine was frequently broken and yielded only 45 probable cases out of over 131,000 people quarantined (*10*). However, the suspected case-patients who were admitted to the hospital led to the discovery of many probable SARS case-patients. For most of May, the ratio between the number of probable cases reclassified from suspected cases and those removed from the suspected SARS list was roughly one to one. Therefore, reporting and admitting suspected cases appeared to have worked in identifying SARS cases. Nonetheless, almost 73% of all traceable infections in Taiwan occurred in hospital settings (Chwan-Chuan King, unpub. data). Hence, determining the circumstances under which these infections occurred is of interest.

**Figure 2:**
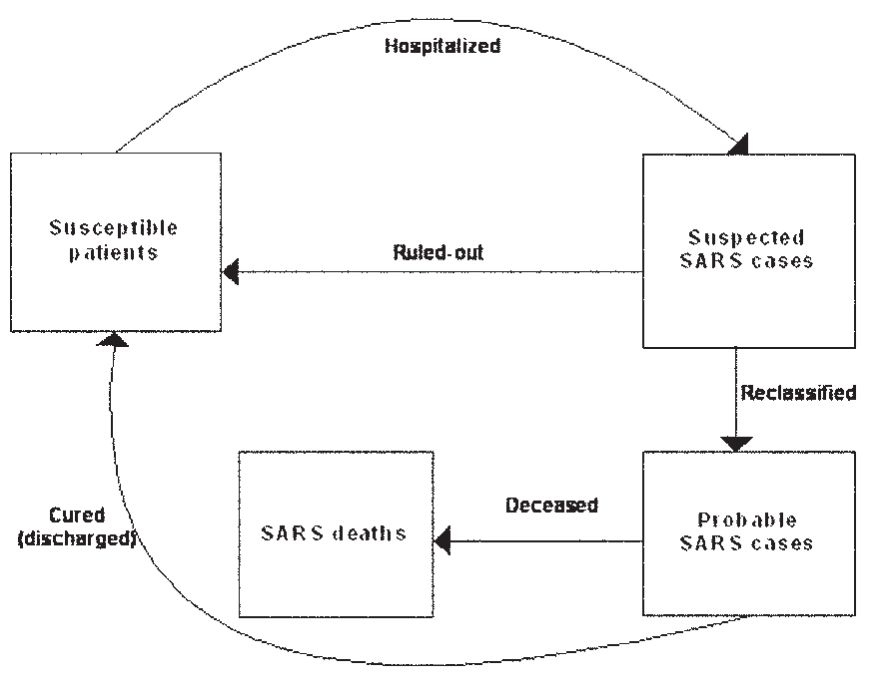
Flow diagram for the model dynamics of the model proposed.

To this end, we considered a model with susceptible patients (S_n_), hospitalized suspected case-patients (H_n_), reported probable SARS case-patients (I_n_), and the accumulated SARS deaths (D_n_). The exposed population was not considered since there had been no documented evidence of transmission before onset of symptoms (*11*). Persons suspected of having SARS were admitted when they had onset of some symptoms combined with a record of recent exposure. Such admission procedures, as well as the protocols for reclassification and downgrading of cases, were carried out in compliance with WHO standards. The flow diagram of the model dynamics is given in Figure 2. The details of the model, including the assumptions made, model equations, and the model parameters, are given in Appendix 1.

We used the daily cumulative numbers of reported suspected cases, probable cases, and deaths from May 5 to June 4 for the true data for the respective numbers for H_n_, I_n_, and D_n_ in our model. We chose the data period May 5–June 4 for expediency: it was the only period when all three numbers could be extracted from the Taiwan Center for Disease Control Web site data. We purposely used the number of probable cases by reporting date instead of by onset date to capture what truly happened clinically and in hospital at various stages of a patient’s clinical progression.

To simplify our estimation procedure, we discarded the time dependence (or subscript n) of each parameter, thus considering the parameters as mean estimates of the variable parameters over the period considered. The model equations were simplified to a linear system of simultaneous difference equations with which data can be easily implemented for the parameter estimation procedure. We used the three-stage least squares (3SLS) procedure commonly used in econometrics, which provides a useful parameter estimation procedure for simultaneous equations (*12*). The details of the estimation method are again given in Appendix 2.

## Results

The parameters estimated, without the subscripts, are: λ and β (the respective admission rates due to contact with probable and suspected case-patients at time n-3); ξ (admission rate due to contact with probable case-patient at time n); α (rule out rate of uninfected hospitalized persons at time n); γ (reclassification rate of suspected SARS case-patients to probable at time n); σ (discharge rate of probable SARS patients at time n); ρ (death rate of probable SARS patients at time n). Note that, by their definitions, α, γ, σ, and ρ are proportions between 0 and 1.

From the estimation results, the contributions of contacts of probable case-patients to the suspected SARS population (λ and ξ) are not significantly different from zero. Hence, almost all SARS-infected persons had symptoms and were admitted before their infections were reclassified from suspected to probable SARS. This finding could indicate efficient admission, slow reclassification process, or a mixture of both. The high percentage of nosocomial infections in Taiwan (73% of all traceable cases) suggests that infection from hospitalized suspected case-patients while they waited to be reclassified (and were subsequently placed in negative-pressure rooms) is a major factor in the spread of disease. Most of the newly admitted suspected case-patients were found by onset of symptoms combined with record of contact with other suspected cases of >3 days before (i.e., H_n-3_). We also attempted to fit the data for possible contacts with I_n-k_ and H_n-k_ for k = 1 to 7 (given that the incubation time has been estimated at 2 to 7 days). Only H_n-3_ turned out to be a significant source of contact for the suspected case-patients. This finding gives a time from infection to onset of >3 days.

**Figure 3:**
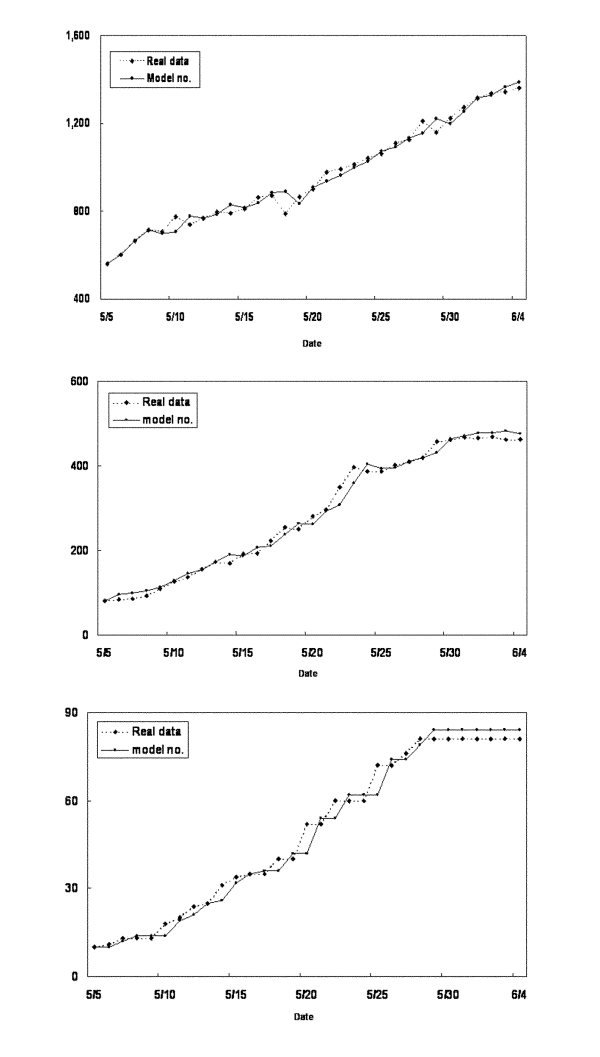
A, number of hospitalized suspected case-patients (H_n_) computed from the model compared with real data from May 5 to June 4, 2003. B, number of reported probable case-patients (I_n_) computed from the model compared with real data from May 5 to June 4. C, cumulative number of deaths due to severe acute respiratory syndrome (D_n_) computed from the model compared with real data from May 5 to June 4.

The results of the parameter estimations are given in Table 1 with the 90% confidence interval (CI) and p value, when appropriate. ρ and β are estimated directly from our estimation procedure of the simultaneous equations with the 90% CI and p values. σ, along with the 90% CI and p value, is obtained through an estimate of 1-ρ-σ; γ is computed from estimate of γδ. α is calculated from the estimate of a product involving δ, γ, and α, from which the 90% CI and p value cannot be easily obtained. The mean proportion of SARS-infected persons among suspected case-patients δ over the period was obtained by using the fact that during the period observed, 1,175 suspected cases were under review. Of these, 562 were reclassified as probable and 613 removed from the category of suspected cases. So we let δ = 562/1175 = 0.4783. The p values indicate that the quality of model fit is good. The numbers computed from the model were plotted against the real data in Figure 3A-C.

**Table 1:** The model parameter values with 90% confidence interval (CI) and p values, when appropriate^a^

Parameter	Estimated value	90% CI	p value
SARS^b^ death rate	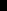 =0.0062	0.0023 to 0.00101	0.0125
Discharge rate of probable case-patients	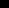 =0.0747	0.000^c^ to 0.1500	<0.0001^d^
Admission rate of suspected case-patients	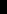 =0.3370	0.0814 to 0.5927	0.0336
Reclassification rate from suspected to probable case	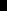 =0.0797	0.0281 to 0.1311	0.0142^e^
Rule-out rate of suspected cases	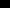 =0.4271	0.3571 to 0.5927	-
Proportion of probable cases in suspected class	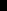 =0.4783	-	-

^a^All rates are per day. ^b^SARS, severe acute respiratory syndrome. ^c^Max{0,-0.0046}. ^d^p value for 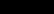. ^e^p value for 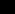.

To make the results more transparent, we used the mean estimates of daily rates to calculate the mean interval for progression through various stages, given in Table 2. The time from admission to reclassification as a probable case is estimated as 1/γ; time from admission to removal from suspected SARS case list is 1/α; time for classification as a probable case to death is 1/ρ multiplied by 0.15, the overall case-fatality rate of SARS patients, as estimated by WHO; the time from probable case to discharge is 1/α multiplied by 0.85, the cure rate.

**Table 2:** Estimated intervals of epidemiologic importance for SARS outbreaks, Taiwan, May 5**–**June 4, 2003^a^

Interval for:	Mean estimate (days)
Admission to reclassification as probable case-patient	12.56
Admission to removal from suspected case-patient category	2.11
Probable case classification to death	24.31
Probable case classification to discharge	11.38

^a^SARS, severe acute respiratory syndrome.

## Discussion

In our study, the gap between mean time from admission to reclassification as probable SARS case-patient was 12.56 days; and the mean time from admission to a case’s being ruled out as a SARS case was 2.11 days. When first admitted with symptoms, a patient is treated with an antimicrobial drug. When the symptoms subsequently subside, the patient status is usually downgraded and the patient is removed from the category of suspected SARS case-patients after a few days of observation. Moreover, anyone who is symptomatic, had contact with this person, but shows no lingering symptoms will also be subsequently quickly downgraded. Hence, a mean estimate of 2.11 days from admission to being ruled out as a case seems reasonable. On the other hand, if the antimicrobial treatment does not yield marked improvement, a person is kept under observation for >7 days, when either lung x-rays or other tests (antibody test or polymerase chain reaction) will determine if the patient’s case should be reclassified as probable SARS. The mean of 12.56 days suggests some delay, either in the cross-checking of diagnostic test results or in the reporting procedure. Confusion regarding case definition and diagnostic procedure (*13*) might also contribute to the delay. The mean time from classification of a case as probable to death is 24.31 days, implying a mean admission to death time of 36.87 days. The estimate is slightly higher than that for Hong Kong estimated by Donnelly et al. (*14*) (Table 3). However, this quantity is highly correlated to how quickly a person with onset of symptoms is admitted. As demonstrated with the Hong Kong data (*14*), the maximum likelihood mean time from onset to admission decreased as the epidemic progressed, probably reflecting a heightened alertness in the general public as well as the health profession. Given the near-panic in Taipei evident from the end of April to most of May, many infected persons (and many non-SARS patients as well) were reported and admitted quickly. However, the fact that most of the infections had occurred in hospital settings highlights the inadequacies in hospital management during this period to effectively isolate suspected SARS case-patients, and instead allowing the spread of SARS to medical staff, other patients, and visitors to the hospital wards.

**Table 3:** Comparison of the estimated intervals from admission to death or discharge for SARS patients in Taiwan with those from Hong Kong study^a^

	Days	
Interval for:	Taiwan	Hong Kong
Admission to designation as a probable case-patient to death	36.87	35.9
Admission to designation as a probable case-patient to discharge	23.94	23.5

^a^By Donnelly et al. (*13*). SARS, severe acute respiratory syndrome.

The total time from admission to discharge for a SARS patient was 23.94 days. To obtain a “mean effective reproductive number for the observed time period,” R*, we use the mean admission rate by suspected cases (β) and multiply it by the mean time the person spent as a suspected case-patient before reclassification (12.56 days) to get R* = 4.23. However, this figure might be an overestimate because of uncertainty regarding how infectious a SARS patient is, relative to the change in his or her viral load (*15*). Note also that the term “mean” refers to averaging over the observed period, to distinguish from the effective reproductive number at time *t*, R_t_ (*6*,*7*). Figure 1 shows the increases of probable cases in the first 20 days of the period considered, followed by a leveling off of cases. Since β is the effective infection rate of one SARS patient (and also the product of effective contact rate and transmission probability per contact), three factors stood out as critical to any control measure for a SARS outbreak: 1) effective isolation of admitted patients to decrease contact rate, 2) improved safety precautions for hospital staff to lower transmission probability in case of close contact, and 3) shortened reclassification time so that the probable cases-patients can be identified swiftly and put in negative-pressure isolation rooms. A breakdown in any of these measures would lead to temporary failure of the whole system, as witnessed in the outbreak in Taiwan.

## Conclusion

The results for the mean effective reproductive number, R*, suggest that the easiest way to reduce infections is more efficient diagnosis of the probable SARS case-patients and their speedy isolation in negative-pressure rooms. In light of the present lack of accurate diagnostic testing for SARS, public health measures aimed at more efficient clinical diagnosis, isolation of suspected case-patients, and reclassification procedures could greatly reduce the number of infections in future outbreaks. Such steps could be accomplished by quickly identifying the true suspected SARS cases, speedy reporting, effective in-hospital isolation, and fast reclassification of the SARS patients.

The quarantine implemented in Taiwan resulted in only a small number of persons later diagnosed as suspected or probable case-patients. However, one can only speculate about the number of additional infections that the quarantine of these few patients prevented. Events in Canada, for example, demonstrated how one misreported case could lead to an entirely new wave of infections. While there is ample evidence that the quarantine implemented by several countries was instrumental in stopping the spread of SARS, the important public health policy decision of using quarantine as an intervention measure, weighed against its socioeconomic costs, requires further studies with better data and more detailed mathematical modeling.

We had attempted to obtain the estimates by splitting the observed time period into two distinct intervals to see if the three factors involved indeed show a decrease during the course of the observed period. Unfortunately, limited data size inhibits such an endeavor. With the help of Center for Disease Control of Taiwan, more extensive data are currently being collected and generated, including information on the chains of infections as well as clusters. Such data collection takes time, involving the difficult task of contact tracing, but it will form the basis of a more comprehensive modeling study in the future, one that can account for the complete sequence of events.

From the model, it is also clear that the estimated parameters should be time-dependent. However, given the limited data available, one must make simplifications to estimate the means of the parameters over the observed period. With more and better data, one could perhaps estimate the parameters over smaller periods of interest during the complete progression of the epidemic, if not the parameter values for each time n.

Another crucial factor in the outbreak is spatial heterogeneity (i.e., diversity in spatial dimension, brought on by the factor of distance). As Hoping Hospital was closed on April 24 in the aftermath of cluster infections, its patients were allowed to disperse freely to other hospitals; some transferred though the medical system, others on their own. This dispersal of infected persons was directly responsible for several hospital cluster infections in Taipei and even one in Kaohsiung, the southern port city, the effect of which cannot be examined without introducing spatial heterogeneity into the model. Dye and Gay (*8*) have presented a lucid argument for the confounding role of heterogeneity in epidemic models. Heterogeneity, regardless of whether in host, transmission, spatial, or any other form, cannot be easily conveyed in a complicated general model. One needs to design specific models with a specifically generated dataset to address specific situations. The spread of SARS thus far has been highly society-dependent: under different social settings, SARS has gained foothold in each country or region in a different way, albeit only shortly, be it Hong Kong, Singapore, Toronto, China, or Taiwan. As a long-term goal, to achieve global eradication of the SARS-CoV, one must understand each distinct pattern of transmission, perhaps by distinct and specific SARS modeling.

## Supplementary Material

Appendix 1 The Model

Appendix 2 Estimation Method
