# Supplementary material for: SARS Outbreak, Taiwan, 2003
Source: Emerg Infect Dis. 2004 Feb;10(2):201–6. doi: 10.3201/eid1002.030515 (PMC3322921; doi:10.3201/eid1002.030515)
Supplement: Appendix 1 — The Model [file 03-0515-app1-s1.pdf]

Appendix 1. The Model

Model Variables

S_n – The number of susceptible persons at time t = n.

H_n – The number of hospitalized suspected case-patients at time t = n.

I_n – The number of living probable SARS case-patients at time t = n.

D_n – The cumulative number of SARS deaths at time t = n.

Note that time unit is in days.

Assumptions

1. A person is moved out of susceptible class only after onset of symptoms and/or having a close contact with a probable case-patient.
2. An infective person can infect others at either suspected or probable stages.
3. A hospitalized suspected case-patient is removed from the suspected class either by reclassification to a probable SARS case-patient or by returning to susceptible class with no immunity. (If there is immunity, one can always add a new class of persons with immunity. For the present model this assumption is not important for our estimation result.)

Parameters

l_n – Admission rate due to contact with probable SARS case-patient at time n–3.

b_n – Admission rate due to contact with suspected case-patient at time n–3.

x_n – Admission rate due to contacts with probable case-patient at time n.

a_n – Rule-out rate of uninfected hospitalized persons at time n.

g_n – Reclassification rate of suspected SARS case-patients to probable at time n.

s_n – Discharge rate of probable SARS patients at time n.

r_n – Fatality rate of probable SARS patients at time n.

d_n – Proportion of infected persons among all suspected case-patients at time n.

Note that a_n, g_n, s_n, r_n, and d_n are proportions between 0 and 1.

The model equations, which describe the change in the model variables from time n to n+1, are as follows:

$$S_{n+1} = S_n - \lambda_n I_{n-3} - \beta_n H_{n-3} - \xi_n I_n + \alpha_n(1-\delta_n)H_n + \sigma_n I_n$$
$$H_{n+1} = \lambda_n I_{n-3} + \xi_n I_n + \beta_n H_{n-3} + (1-\gamma_n)\delta_n H_n + (1-\alpha_n)(1-\delta_n)H_n$$
$$I_{n+1} = I_n - (\sigma_n + \rho_n)I_n + \gamma_n \delta_n H_n$$
$$D_{n+1} = D_n + \rho_n I_n$$

with

$$S_{n+1} + H_{n+1} + I_{n+1} + D_{n+1} = S_n + H_n + I_n + D_n .$$

The flow diagram for the dynamics is given in Figure 2.

Since the equations for H_{n+1}, I_{n+1} and D_{n+1} involve only H_n, I_n and D_n, we can consider these three equations in a simple model

$$H_{n+1} = \lambda_n I_{n-3} + \xi_n I_n + \beta_n H_{n-3} + [(1-\gamma_n)\delta_n + (1-\alpha_n)(1-\delta_n)]H_n$$
$$I_{n+1} = (1-\sigma_n-\rho_n)I_n + \gamma_n \delta_n H_n$$
$$D_{n+1} = D_n + \rho_n I_n$$

which can be put in the following matrix form:

$$\begin{bmatrix} H_{n+1} \\ I_{n+1} \\ D_{n+1} \end{bmatrix} = \begin{bmatrix} (1-\gamma_n)\delta_n + (1-\alpha_n)(1-\delta_n) & \xi_n & 0 \\ \gamma_n \delta_n & (1-\sigma_n-\rho_n) & 0 \\ 0 & \rho_n & 1 \end{bmatrix} \begin{bmatrix} H_n \\ I_n \\ D_n \end{bmatrix} + \begin{bmatrix} \beta_n & \lambda_n & 0 \\ 0 & 0 & 0 \\ 0 & 0 & 0 \end{bmatrix} \begin{bmatrix} H_{n-3} \\ I_{n-3} \\ D_{n-3} \end{bmatrix}$$

The data for H_n, I_n, and D_n, the respective numbers of admitted suspected case-patients, reported probable SARS case-patients, and SARS deaths, are available for parameter estimation.
