# Supplementary material for: SARS Outbreak, Taiwan, 2003
Source: Emerg Infect Dis. 2004 Feb;10(2):201–6. doi: 10.3201/eid1002.030515 (PMC3322921; doi:10.3201/eid1002.030515)
Supplement: Appendix 2 — Estimation Method [file 03-0515-app2-s2.pdf]

Appendix 2. Estimation Method

We treat the linear system of equations above as a multiequation simulation model, which allows us to account for the interrelationship within a set of variables, namely, H_n, I_n, and D_n, which are called endogenous variables in econometrics (11). Two-stage least squares (2SLS) and 3SLS can both provide a very useful estimation procedure for simultaneous equation. However, 2SLS is inefficient when the system of equations contains lagged dependent variables, which account for adjustments that take place over time. We can achieve a gain in efficiency by applying 3SLS. It involves applying generalized least squares estimation to a system of equations, each of which has first been estimated using 2SLS. The 3SLS procedure yields more efficient parameter estimates than does 2SLS because it takes into account the cross-equation correlation.
